# Blood Lead Concentrations and Antibody Levels to Measles, Mumps, and Rubella among U.S. Children

**DOI:** 10.3390/ijerph16173035

**Published:** 2019-08-22

**Authors:** Todd A. Jusko, Kyra Singh, Elizabeth A. Greener, Marina Oktapodas Feiler, Kelly Thevenet-Morrison, B. Paige Lawrence, Robert O. Wright, Sally W. Thurston

**Affiliations:** 1 Department of Public Health Sciences, University of Rochester School of Medicine and Dentistry, Rochester, NY 14642, USA; 2 Department of Environmental Medicine, University of Rochester School of Medicine and Dentistry, Rochester, NY 14642, USA; 3 Department of Biostatistics and Computational Biology, University of Rochester School of Medicine and Dentistry, Rochester, NY 14642, USA; 4 Department of Environmental Medicine and Public Health, Icahn School of Medicine at Mount Sinai, New York, NY 10029, USA

**Keywords:** lead, NHANES, MMR, vaccine, vaccination, immunotoxicology, immune, immunology, B cell, T cell, seronegative, seropositive, herd immunity

## Abstract

Child blood lead concentrations have been associated with measures of immune dysregulation in nationally representative study samples. However, response to vaccination—often considered the gold standard in immunotoxicity testing—has not been examined in relation to typical background lead concentrations common among U.S. children. The present study estimated the association between blood lead concentrations and antigen-specific antibody levels to measles, mumps, and rubella in a nationally representative sample of 7005 U.S. children aged 6–17 years. Data from the 1999–2004 cycles of the National Health and Nutrition Examination Survey (NHANES) were used. In the adjusted models, children with blood lead concentrations between 1 and 5 µg/dL had an 11% lower anti-measles (95% CI: −16, −5) and a 6% lower anti-mumps antibody level (95% CI: −11, −2) compared to children with blood lead concentrations <1 µg/dL. The odds of a seronegative anti-measles antibody level was approximately two-fold greater for children with blood lead concentrations between 1 and 5 µg/dL compared to children with blood lead concentrations <1 µg/dL (OR = 2.0, 95% CI: 1.4, 3.1). The adverse associations observed in the present study provide further evidence of potential immunosuppression at blood lead concentrations <5 µg/dL, the present Centers for Disease Control and Prevention action level.

## 1. Introduction

Experimental studies in animal models have demonstrated that lead exposure causes a dysregulation of the immune system that affects both Th1 and Th2 cell-mediated responses [1]. This dysregulation is hypothesized to have downstream effects on immune function, including attenuated antibody responses to antigen, greater infectious morbidity, and increased hypersensitivity. Recent studies conducted using data from the National Health and Nutrition Examination Survey (NHANES) support these experimental findings. For example, positive associations between higher blood lead concentrations and nasal colonization with methicillin-resistant *Staphylococcus aureus* (MRSA) and seropositivity for *Helicobacter pylori*, *Toxoplasma gondii*, and Hepatitis B virus (HBV) infections have been observed [2,3], and associations between blood lead concentrations and *Helicobacter pylori* and *Toxoplasma gondii* were strongest amongst participants younger than 13 years of age [2]. Studies utilizing NHANES data have also observed evidence of Th2-type immune dysregulation. For instance, in another analysis of NHANES data from 2005 to 2006, each 1 µg/dL of blood lead was associated with an 11% increase in total circulating IgE concentrations among children 2–12 years of age [4]. 

The present study builds on these findings of lead-associated immune dysregulation observed in NHANES, focusing on T cell-dependent antibody responses. T cell-dependent antibody responses are the “gold standard” outcome when assessing potential immunotoxicity in experimental and human studies, since optimal responses require the proper functioning of multiple immune cell types (e.g., antigen presenting cells, B cells, and T cells) [5,6,7,8,9,10,11]. Despite their importance, lead-associated changes in antibody response have not been examined in NHANES. A recent National Toxicology Program (NTP) report concluded that there is limited evidence that blood lead concentrations <10 µg/dL are associated with adverse immune effects in children, largely due to the lack of human studies examining antibody response [12]. The present study aims to help fill this research gap.

## 2. Materials and Methods

NHANES data from the 1999–2004 cycles were utilized. Other cycles were considered, but omitted because of incomplete antibody data or varying antibody measurement units that could not be re-scaled. NHANES is a series of 2-year cross-sectional, stratified, nationally representative multistage probability surveys of the civilian U.S. population. All the protocols were approved by the research ethics boards of the National Center for Health Statistics. Whole-blood lead concentrations were determined using atomic absorption spectroscopy (1999–2002) or inductively coupled plasma mass spectrometry (2003–2004) by the U.S. Centers for Disease Control and Prevention (CDC). The limit of detection (LOD) for blood lead concentrations was 0.3 μg/dL (1999–2002) and 0.28 μg/dL (2003–2004) [13]. All concentrations below the LOD were substituted by the CDC, with the LOD divided by the square root of 2. Between 1999 and 2004, the MMR (meales, mumps, rubella) vaccine schedule was a first dose at 12–18 months and a second dose at 4–6 years, with a catch-up second dose by 11–12 years of age [14]. Immunoglobulin G levels to measles and rubella antigens were determined by standardized ELISA protocols developed by the Immunoserology Unit of the California State Department of Health Services (CSDHS), Viral and Rickettsial Disease Laboratory (VRDL). Immunoglobulin levels specific to mumps antigens were determined by the Wampole Mumps IgG Test (Ref. # 425901CE). Seropositive values were ≥1.0 for anti-measles, ≥1.1 for anti-mumps, and ≥1.0 to <10.0 for anti-rubella. Anti-mumps antibody levels between 0.901 and 1.099 were considered indeterminate and anti-rubella levels ≥10 were indicative of infection.

Analyses included data from participants 6 to 17 years of age. Descriptive statistics were computed using the SURVEYFREQ procedure and multivariable models were fit utilizing the SURVEYREG and SURVEYLOGISTIC procedures in SAS software (version 9.4; SAS Institute Inc., Cary NC USA). Regression models included interview sample weights and survey design variables to account for differential probabilities of non-response, non-coverage, selection, and sample design to obtain representative estimates [15]. The covariates child sex, age (in months), race/ethnicity (Mexican American, other Hispanic, Non-Hispanic White, Non-Hispanic Black, and other race, including multi-racial), family poverty to income ratio (continuous), and NHANES cycle (1999–2000, 2001–2002, 2003–2004) were included in all statistical models as potential confounders. Logisitic models were fit comparing seronegative to seropositive antibody levels, where seropositive antibody levels were the reference category. For anti-mumps antibody logistic models, three logistic models were fit comparing 1) seronegative to seropositive antibody levels (excluding indeterminate antibody levels), 2) seronegative to seropositive and indeterminate antibody levels, and 3) seronegative and indeterminate antibody levels to seropositive antibody levels. Polychlorinated biphenyl (PCB) and perfluorooctanoic and perfluorooctanesulfonic acid (PFOA and PFOS) concentrations were considered as potential confounders, as these compounds have been associated with immunosuppression in children; however, concentrations of PCB-153, PFOA, and PFOS were not associated with child lead concentrations in these data (−0.03 ≤ r ≤ 0.05) [8,16]. Child blood lead concentration was modeled as an untransformed, continuous variable in µg/dL and as a categorical variable (<1; 1<5; ≥5 µg/dL) in separate regression models to assess potential non-linearity and to quantify associations between blood lead concentrations below 5 µg/dL and antibody-specific levels to each vaccine antigen. All antibody outcomes were transformed by the natural log to normalize variance. Participants with seropositive and seronegative antibody levels for each vaccine were included in the primary analyses, consistent with previous studies of environmental exposures and vaccine antibody levels [8,11]. As a secondary analysis, only seropositive antibody-specific levels were included in the linear models of the lead–antibody associations. 

Two additional models were fit for both linear and logistic models (1) excluding children with blood lead concentrations ≥10 µg/dL and (2) modeling the child’s blood lead concentration utilizing a natural log transform in order to reduce the influence of potential outliers.

## 3. Results

In total, data from 7005 children were available for inclusion in analyses. Child age was uniformly distributed across the 6- to 17-year-old sample, with approximately 7–9% of children in each 1-year age group (data not shown in tabular form). Blood lead concentrations averaged 1.4 µg/dL (median = 1.0 µg/dL); 97.8% of children had concentrations below 5 µg/dL and 99.7% had concentrations below 10 µg/dL. The highest three concentrations were 57.1, 34.8, and 16.1 µg/dL. Less than 1% of blood lead measurements were below the LOD.

Overall, for models that included seropositive and seronegative antibody levels, each 1 µg/dL difference in blood lead concentration was associated with a 2%–3% lower antibody-specific level for anti-measles and anti-mumps, while the dose-response for anti-rubella was inconsistent (Table 1). In categorical analyses, children with blood lead concentrations between 1 and 5 µg/dL had an 11% lower anti-measles (95% CI: −16, −5) and a 6% lower anti-mumps level (95% CI: −11, −2) compared to children with blood lead concentrations <1 µg/dL. Children with blood lead concentrations of 5 µg/dL or greater had 21% lower anti-measles levels (95% CI: −35, −3) and 19% lower anti-mumps levels (95% CI: −30, −6) compared to children with blood lead concentrations <1 µg/dL. 

Models fit utilizing only seropositive antigen-specific antibody values (Table 2) showed some attenuation. When modeled as an untransformed, continuous variable, each 1 µg/dL difference in blood lead was associated with a 2% lower anti-measles antibody level and a 0.5% lower anti-mumps antibody level (Table 2). Modeled categorically, children with blood lead concentrations between 1 and 5 µg/dL had a 5% lower anti-measles (95% CI: −10, −0.5) and a 1% lower anti-mumps level (95% CI: −5, 2) compared to children with blood lead concentrations <1 µg/dL.

Logistic models estimating the odds of seronegativity indicated increased odds of seronegative antibody levels for both anti-measles and anti-mumps, but not for anti-rubella antibody level models (Table 3). The odds of a seronegative anti-measles antibody level was approximately 2 times greater for children in the 1<5 µg/dL category relative to the <1 µg/dL category (OR = 2.0, 95% CI: 1.4, 3.1) and 2.6 times greater for children in the ≥5 µg/dL category relative to the <1 µg/dL category, though the confidence limits were wide (OR = 2.6, 95% CI: 0.4, 16.7). For anti-mumps antibody levels, the odds of seronegativity were approximately 1.6 times greater for children in the 1<5 µg/dL category relative to the <1 µg/dL category and between 2.6 and 3.5 times greater for children in the ≥5 µg/dL category relative to the <1 µg/dL category, depending on whether or where indeterminate antibody levels were included (Table 3). When blood lead concentration was entered as a continuous variable, the odds of seronegativity increased with increasing blood lead concentrations, consistent with the results from models fit with categorical lead variables.

Analyses excluding children with blood lead concentrations ≥10 µg/dL resulted in slightly stronger, but not meaningfully different estimates of association in both linear and logistic regression models (Table 1, Table 2 and Table 3). Similarly, modelling natural log transformed blood lead concentrations resulted in stronger effect estimates for anti-measles and anti-mumps antibody levels compared with untransformed continuous models of blood lead concentration for both linear and logistic models (Table 1, Table 2 and Table 3).

## 4. Discussion

In this nationally representative cross-sectional study, higher blood lead concentrations in children aged 6–17 years were associated with lower circulating levels of anti-measles and anti-mumps antibodies. Higher blood lead concentrations were also associated with greater odds of a seronegative antibody level, compared with lower levels of blood lead. The associations observed in the present study suggest potential immunosuppression even at blood lead levels that are less than 5 µg/dL, which is the current CDC reference level for blood lead [17]. Specifically, the odds of having a seronegative anti-measles antibody level was approximately two times greater for children with blood lead concentrations between 1 and 5 µg/dL compared to children with blood lead concentrations <1 µg/dL, and approximately 1.6 times greater for anti-mumps antibody levels.

Higher blood lead concentrations were associated with lower levels of anti-measles and anti-mumps antibodies in the present study. As noted in a recent review and in a report from the U.S. National Toxicology Program [12,18], there is a paucity of data regarding lead exposure and immune outcomes in children. Consequently, the findings from the present study are challenging to integrate into the current literature on lead immunotoxicity. To our knowledge, one other study examined lead exposure in relation to antigen-specific antibody levels. In that cross-sectional study of 279 infants aged 9–48 months, weak correlations were observed between blood lead levels and post-vaccination rubella titers (r = −0.09), and results were not adjusted for potential confounding factors [19]. This essentially null finding is consistent with the results observed in the present study, where blood lead concentrations in 6–17-year-old children were not associated with rubella-specific antibody levels, although antibody-specific responses to measles and mumps were not measured in the previous study. 

In general, the potential effects of lead exposure on the developing human immune system are not well understood, and most previous studies have focused on levels of circulating immune cell populations (i.e., CD3, CD4, CD8, and B cells) [19,20,21,22,23,24]. For example, three studies from a cohort of preschool aged children in China examined high blood lead levels (≥10 µg/dL) versus low blood lead levels (<10 µg/dL) in relation to measures of immune function [21,22,23]. Overall, reductions in mean lymphocyte levels were observed among the group with higher blood lead levels compared with lower levels [22] and inverse correlations between blood lead and T-lymphocyte levels were observed [21]. Further, among females in this cohort, lower IgG and higher IgE among the high lead, versus low lead group were observed [23]. Another study of 1561 children observed no evidence of association between blood lead concentrations and immune cell populations among children aged 3 years and older [24], however, among younger children, the authors observed higher IgA, IgG, IgM, and B cell concentrations among those with a blood lead levels ≥15 µg/dL [24]. A study of 331 German children aged 7–10 years observed fewer T cells, cytotoxic T cells, and B cells with higher blood lead concentrations [20]. At the same time, some of the above studies have also reported outcomes suggestive of hypersensitivity (i.e., increases in IgE, and interleukin-4 (IL-4)), with higher blood lead levels [19,20,23,24], although results from the large MIREC cohort were not suggestive of an association between maternal lead concentrations and cord blood interleukin-33 (IL-33) or IgE [25]. While these studies have informed the field, it is still unclear whether differences in these cell populations impact the functioning of the immune system [1].

In the present study, the lead–antibody associations estimated from linear models were attenuated when seronegative antibody-specific antibody levels were excluded. An argument for the exclusion of seronegative antigen-specific antibody values is that exclusion decreases the likelihood of unvaccinated children in the sample, ergo increasing the likelihood that antibody-specific antibody levels of study particpants are the result of vaccination, and not a result of naturally acquired immunity via infection [26]. However, studies have demonstrated that IgG-specific antibodies to measles and mumps, in the absence of additional information, are not always indicative of vaccination rather than natural infection. For instance, antigen-specific IgG antibody levels to measles were higher among children with naturally acquired infections compared with vaccinated children in a study of children 12 years of age, and mumps-specific anti-IgG levels among 18-29 year old Czech adults tended to be higher, not lower, among those with previous natural infection versus those previously vaccinated [27,28]. These findings suggest that antibody-specific levels of IgG for anti-measles and anti-mumps are not a useful metric for determining vaccination status without additional information, and therefore, seronegative antibody levels should not be immediately excluded from studies assessing antigen-specific IgG levels of circulating antibodies.

The associations between blood lead concentrations and anti-measles and anti-mumps antibodies were robust to sensitivity analyses which excluded blood lead concentrations greater than 10 **μ**g/dL or respecified the exposure variable as the natural log of the blood lead concentration. In general, the estimated association between blood lead and antibody-specific antibody levels was stronger when excluding blood lead concentrations ≥ 10 μg/dL than when these concentrations were included in statistical models. However, the natural log results assume a curvilinear dose response between blood lead concentration and antibody level, and this curvilinear model may not hold for blood lead concentrations ≥ 10 μg/dL for which data were sparse in this sample.

The present study has some limitations that deserve mention. First, data from NHANES are not longitudinal and therefore do not permit multiple assessments of either blood lead concentrations or of antigen-specific antibody levels throughout childhood. The effect of modeling a single concurrent blood lead as opposed to an integrated average of previous exposure likely introduces non-differential exposure misclassiciation, biasing estimated blood lead–antibody associations towards the null. Unmeasured positive confounding might also have biased the estimated associations towards the null [29]. An unmeasured positive confounder would have to be positively associated with exposure and negatively associated with the outcome, or vice versa. Preliminary models considered variables such as environmental tobacco smoke and child BMI as potential confounders, but inclusion of these variables tended to move the estimated lead–antibody associations further away from the null hypothesis (data not shown). Finally, the antigen-specific IgG antibody levels reported by NHANES and utilized in the present analysis are not necessarily the result of vaccination or the same number of vaccinations in each child. Rather, detectible antibody levels could be the result of a natural infection, a single vaccination administered 2 years prior to the study blood draw, or a booster vaccine received 2 months prior to the study blood draw [14]. For this limitation to explain the observed results, recent vaccination (resulting in higher antigen-specific antibody levels) would have to be related to lower blood lead levels in this age group. Since antibody levels and blood lead concentrations wane with age in this group, this scenario seems unlikely [13,30,31].

In addition to using nationally representative data from the 1999–2004 NHANES, the present study included approximately 7000 6- to 17-year-old children. The large sample size served to decrease the effects of random error and chance findings in the study. This study also included antibody levels to three live-attenuated vaccines administered on the same 12–18 month and 4–6 year schedule during the study period. Compared with non-live vaccines, live vaccines like the MMR vaccine induce a more intense innate immune response, eliciting a greater number of effector mechanisms, resulting in greater antibody proliferation and, theoretically, a more prolonged antibody persistence [32]. Thus, if exposure to lead indeed causes immune dysfunction, then the comprehensive nature of the immune response to MMR increases the likelihood of detecting an association. Finally, sensitivity analyses demonstrated that the results of the present study are unlikely due to influential lead values or to how the lead exposure variable was paramterized in statistical models.

## 5. Conclusions

In experimental studies, continuous low-level exposure can impact the developing immune system, especially during a critical windows of immune development, such as during gestation and childhood [6,33]. The results of the present nationally representative study suggest that blood lead concentrations below the current CDC reference level for blood lead adversely impact immune function, as measured by circulating antigen-specific antibody levels to measles, mumps, and rubella viruses. These findings have public health implications, given that the blood lead concentrations observed in this study are directly generalizable to the United States pediatric population. Furthermore, the results indicate that the odds of seronegative anti-measles and anti-mumps antibody levels are approximately 1.5 to 2 times greater among children with blood lead concentrations between 1<5 µg/dL (below the CDC reference level), compared to children with blood lead concentrations <1 µg/dL. If causal, decreases in anti-measles and anti-mumps seropositivity would reduce herd immunity and increase the risk of infection and outbreak, particularly in geographic areas with already lowered herd immunity to these antigens [30,31]. 

## Figures and Tables

**Table 1:** Adjusted ^1^ percent change in anti-measles, anti-mumps, and anti-rubella levels in relation to child blood lead concentration.

	Anti−Measles				Anti−Mumps				Anti−Rubella			
Blood Lead Measure	N	% Diff ^2^	95% CI ^3^	*p* Value	N	% Diff	95% CI	*p* Value	N	% Diff	95% CI	*p* Value
Categorical												
<1 µg/dL	2535	Ref	-	-	2531	Ref	-	-	2535	Ref	-	-
1<5 µg/dL	4318	−10.89	(−16.35, −5.06)	0.001	4306	−6.28	(−10.53, −1.82)	0.007	4318	−7.97	(−13.32, −2.29)	0.008
≥5 µg/dL	152	−20.81	(−35.15, −3.30)	0.023	154	−18.54	(−29.59, −5.75)	0.007	152	−1.93	(−28.88, 35.25)	0.904
Linear (per 1 µg/dL) ^4^	7005	−2.75	(−5.07, −0.38)	0.025	6991	−2.07	(−3.87, −0.24)	0.028	7005	−0.0007	(−2.58, 2.65)	0.999
Linear (per 1 µg/dL) ^5^	6992	−3.64	(−6.07, −1.15)	0.005	6978	−2.41	(−4.32, −0.46)	0.017	6992	−0.31	(−3.15, 2.62)	0.831
Linear (per 1 log_e_) ^6^	7005	−8.58	(−12.94, −3.99)	0.001	6991	−5.61	(−8.74, −2.38)	0.001	7005	−1.87	(−7.08, 3.63)	0.489

^1^ Adjusted for child sex, age (months), race/ethnicity (5 categories), family poverty to income ratio, NHANES cycle (1999–2000, 2001–2002, 2003–2004); ^2^ Percent difference in antibody level for each increment of exposure; ^3^ 95% confidence interval; ^4^ Linear regression model with blood lead modelled as a continuous, untransformed concentration; ^5^ Linear regression model with blood lead modelled as a continuous, untransformed concentration, excluding blood lead concentrations ≥10 µg/dL; ^6^ Linear regression model with blood lead modelled as a continuous concentration with log_e_ transformation.

**Table 2:** Adjusted ^1^ percent change in seropositive anti-measles, anti-mumps, and anti-rubella levels in relation to child blood lead concentration.

	Anti−Measles				Anti−Mumps				Anti−Rubella			
Blood Lead Measure	N	% Diff ^2^	95% CI ^3^	*p* Value	N	% Diff	95% CI	*p* Value	N	% Diff	95% CI	*p* Value
Categorical												
<1 µg/dL	2634	Ref	-	-	2519	Ref	-	-	2581	Ref	-	-
1<5 µg/dL	4570	−5.49	(−10.26, −0.46)	0.034	4332	−1.37	(−4.80, 2.19)	0.437	4510	−2.90	(−7.16, 1.56)	0.194
≥5 µg/dL	163	−17.42	(−30.62, −1.71)	0.032	154	−6.40	(−19.99, 2.19)	0.400	164	0.76	(−25.75, 36.74)	0.961
Linear (per 1 µg/dL) ^4^	6832	−2.18	(−4.22, −0.09)	0.041	6492	−0.46	(−1.30, 0.38)	0.273	6719	0.42	(−2.19, 3.10)	0.751
Linear (per 1 µg/dL) ^5^	6819	−2.39	(−4.66, −0.08)	0.043	6480	−0.40	(−2.07, 1.31)	0.638	6707	0.71	(−2.27, 3.78)	0.638
Linear (per 1 log_e_) ^6^	6832	−5.51	(−9.90, −0.90)	0.021	6492	−1.42	(−3.70, 0.91)	0.224	6719	1.00	(−3.86, 6.11)	0.686

^1^ Adjusted for child sex, age (months), race/ethnicity (5 categories), family poverty to income ratio, NHANES cycle (1999–2000, 2001–2002, 2003–2004); ^2^ Percent difference in antibody level for each increment of exposure; ^3^ 95% confidence interval; ^4^ Linear regression model with blood lead modelled as a continuous, untransformed concentration; ^5^ Linear regression model with blood lead modelled as a continuous, untransformed concentration, excluding blood lead concentrations ≥10 µg/dL; ^6^ Linear regression model with blood lead modelled as a continuous concentration, with log_e_ tranformation.

**Table 3:** Adjusted ^1^ odds of a negative versus positive anti-measles, anti-mumps, and anti-rubella level in relation to child blood lead concentration.

	Anti-Measles ^2^		Anti-Mumps		Anti-Rubella
	Neg vs. Pos	Neg vs. Pos	Neg vs. Pos and Ind	Neg and Ind vs. Pos	Neg vs. Pos
	(*n =* 7005)	(*n =* 6786)	(*n =* 6991)	(*n =* 6991)	(*n =* 7005)
Blood Lead Measure	OR (95% CI) ^3^	OR (95% CI)	OR (95% CI)	OR (95% CI)	OR (95% CI)
Categorical					
<1 µg/dL	1.00 (ref)	1.00 (ref)	1.00 (ref)	1.00 (ref)	1.00 (ref)
1<5 µg/dL	2.03 (1.35, 3.05)	1.64 (1.12, 2.40)	1.61 (1.10, 2.37)	1.61 (1.22, 2.12)	1.35 (0.92, 1.98)
≥5 µg/dL	2.64 (0.42, 16.73)	3.46(1.26, 9.48)	3.29 (1.23, 8.80)	2.58 (1.03, 6.50)	0.75 (0.31, 1.79)
Linear (per 1 µg/dL) ^4^	1.05 (0.96, 1.14)	1.10 (0.98, 1.24)	1.10 (0.98, 1.24)	1.08 (0.99, 1.19)	1.00 (0.90, 1.10)
Linear (per 1 µg/dL) ^5^	1.17 (0.98, 1.38)	1.26 (1.10, 1.45)	1.26 (1.10, 1.44)	1.19 (1.06, 1.33)	1.03 (0.86, 1.24)
Linear (per 1 log_e_) ^6^	1.45 (1.12, 1.88)	1.59 (1.18, 2.13)	1.58 (1.18, 2.12)	1.44 (1.15, 1.80)	1.15 (0.83, 1.59)

^1^ Adjusted for child sex, age (months), race/ethnicity (5 categories), family poverty to income ratio, NHANES cycle (1999–2000, 2001–2002, 2003–2004); ^2^ Antibody levels modelled as seronegative (neg) versus seropositive (pos), and for anti-mumps antibody levels, either group also included indeterminate (ind) levels; ^3^ Odds ratio and 95% confidence interval; ^4^ Logistic regression model with blood lead modelled as a continuous untransformed concentration; ^5^ Logistic regression model with blood lead modelled as a continuous, untransformed concentration, excluding blood lead concentrations ≥10 µg/dL; ^6^ Logistic regression model with blood lead modelled as a continuous concentration, with log_e_ transformation.
